# Evaluation of Lung Tumor Target Volume in a Large Sample: Target and Clinical Factors Influencing the Volume Derived From Four-Dimensional CT and Cone Beam CT

**DOI:** 10.3389/fonc.2021.717984

**Published:** 2022-01-20

**Authors:** Fengxiang Li, Tingting Zhang, Xin Sun, Yanlin Qu, Zhen Cui, Tao Zhang, Jianbin Li

**Affiliations:** ^1^ Department of Radiation Oncology, Shandong Cancer Hospital and Institute, Shandong First Medical University and Shandong Academy of Medical Sciences, Jinan, China; ^2^ Department of Biostatistics, School of Public Health, Cheeloo College of Medicine, Shandong University, Jinan, China

**Keywords:** lung tumor, stereotactic body radiation therapy, four dimensional CT, cone beam CT, internal target volume

## Abstract

**Background and Purpose:**

This study aimed to systematically evaluate the influence of target-related and clinical factors on volume differences and the similarity of targets derived from four-dimensional computed tomography (4DCT) and cone beam computed tomography (CBCT) images in lung stereotactic body radiation therapy (SBRT).

**Materials and Methods:**

4DCT and CBCT image data of 210 tumors from 195 patients were analyzed. The internal gross target volume (IGTV) derived from the maximum intensity projection (MIP) of 4DCT (IGTV-MIP) and the IGTV from CBCT (IGTV-CBCT) were compared with the reference IGTV from 10 phases of 4DCT (IGTV-10). The target size, tumor motion, and the similarity between IGTVs were measured. The influence of target-related and clinical factors on the adequacy of IGTVs derived from 4DCT MIP and CBCT images was evaluated.

**Results:**

The mean tumor motion amplitude in the 3D direction was 6.5 ± 5 mm. The mean size ratio of IGTV-CBCT and IGTV-MIP compared to IGTV-10 in all patients was 0.71 ± 0.21 and 0.8 ± 0.14, respectively. Female sex, greater BSA, and larger target size were protective factors, while the Karnofsky Performance Status, body mass index, and motion were risk factors for the similarity between IGTV-MIP and IGTV-10. Older age and larger target size were protective factors, while adhesion to the heart, coexistence with cardiopathy, and tumor motion were risk factors for the similarity between IGTV-CBCT and IGTV-10.

**Conclusion:**

Clinical factors should be considered when using MIP images for defining ITV, and when using CBCT images for verifying treatment targets.

## Introduction

With the advent of the era of systemic targeted and immunotherapy, stereotactic body radiation therapy (SBRT) has not only resulted in favorable antitumor effects for primary early non-small cell lung cancer but also shown significant efficacy in oligometastatic lung tumors (1–3). However, lung SBRT with a combination of targeted or immunotherapy may increase the clinically meaningful risk of pneumonitis (4, 5), which may result in treatment failure. Therefore, more attention should be paid to avoiding the normal tissue being unnecessarily irradiated, when increasing the local control in lung SBRT.

Accurate definition of the characteristics from different simulated images (e.g., conventional three-dimensional CT [3DCT] and four-dimensional CT [4DCT]) and accurate delineation of a reasonable internal target volume (ITV) are preconditions for the success of lung SBRT. A 4DCT scan is considered a reliable tool for simulating respiration-induced intrapulmonary motion (6–9). The individual ITV derived from 4DCT has been widely used in lung SBRT. The volume encompassing the gross tumor volumes (GTVs) delineated on all phases (typically 10 phases) of the 4DCT is accepted as the standard ITV (6–9). However, delineating the GTVs on all phases is time-consuming (10, 11). Maximum intensity projection (MIP) displays the highest density value encountered in each pixel throughout the respiratory cycle of 4DCT (10–14). The MIP is usually used to generate the ITV instead of 10 phases of the 4DCT. However, the use of MIP in clinical practice has caused considerable controversy. Several studies have shown that the MIP might be a reliable tool for target definition (11, 13), while other research has concluded that the MIP underestimates the size of ITV and should not be used in isolation (10, 14, 15). However, these results were obtained based on phantom studies and small patient collectives. There is a lack of comprehensive estimates of the ITV derived from the 4DCT MIP images in studies with large sample sizes.

On-board free-breathing cone beam computed tomography (CBCT) is a useful tool for the target localization of lung tumors (16, 17). The use of CBCT provides a guarantee of precise irradiation during treatment with lung SBRT. Free-breathing CBCT can simulate lung tumor motion, to some extent, and can be used to delineate the online ITV (18–20). Although 4DCBCT has been regarded as a better choice for determination of the ITV during treatment (21, 22), it has not been widely used in the clinical setting and provides poor quality CBCT image sets (23, 24). Previous studies have focused on the differences in size between ITVs derived from 4DCT and CBCT. However, the impact of the target-related and clinicopathologic features on these differences has not been demonstrated completely and systematically (25). CBCT shows an inferior soft tissue contrast compared with CT due to different imaging methods. Moreover, the CBCT target volume might be more easily influenced by the clinicopathologic characteristics of the patient, such as pathological pattern, and Karnofsky Performance Status (KPS) score. Currently, 3DCT is used for conventional fractionation radiation therapy. A thorough understanding of the variation in size between the GTV on 3DCT and the ITV on 4DCT contributes to determining a reliable ITV.

In this study, we assessed the differences in volume and the similarities of the targets derived from 4DCT MIP, CBCT, and 3DCT compared to the ITV derived from 10 phases of 4DCT. The aim was to systematically evaluate the influence of the target-related and clinicopathologic features on these differences in a large sample of patients. Furthermore, we tried to establish a predictive model in relation to the similarity of the targets derived from 4DCT and CBCT based on the significant target-related and clinicopathologic features. To the best of our knowledge, these results have not been evaluated or reported in previous studies. The availability of such information may contribute to a reasonable application of the ITVs derived from 4DCT MIP and CBCT images in clinical practice.

## Materials and Methods

### Patient Selection and Characteristics

This study was a retrospective analysis that was approved by the *Shandong Cancer Hospital and Institute* ethics board, and the need for informed consent from patients was waived. In total, 195 of 438 patients who underwent lung SBRT between May 2015 and December 2019 at the *Shandong Cancer Hospital and Institute* were enrolled. Among the 195 patients, 11 had multiple tumors; this study included a total of 210 tumors. One hundred sixty-two targets were primary lung cancers (146 tumors) and metastases of lung cancer (16 tumors), and 48 were metastases of other solid cancers. All the patients were selected on the basis of the following criteria: 1) peripheral lung tumors or metastases; 2) 4DCT and CBCT images of adequate quality; and 3) GTV that was identifiable on CT images. Patients were excluded if they met the following criteria: 1) 4DCT or CBCT images were missing; 2) the tumors were extensive and diffuse; or 3) the tumor boundary could not be easily distinguished from the surrounding pneumonia.

### CT Simulation and Image Acquisition

All patients were immobilized using vacuum bags or the Body Pro-Lok ONE™ system (CIVCO, Coralville, IA) in the supine position with their arms raised above their head. For each patient, a conventional 3DCT scan of the thoracic region was performed, followed by a 4DCT scan during free breathing on a Brilliance Big Bore CT simulator (Philips Medical Systems, Highland Heights, OH). The 3DCT and 4DCT acquisition protocols have been reported in our previous study (26, 27). The 4DCT images were sorted into 10 bins according to the phase of the breathing signal, with 0% corresponding to end-inhalation and 50% corresponding to end-exhalation. MIPs of the 4DCT data sets were then generated and contained the maximum Hounsfield unit (HU) in each geometric voxel across all time-resolved datasets. The CT images were reconstructed using a thickness of 3 mm or 2 mm (tumors within 1 cm in diameter) and then transferred to the Eclipse treatment planning system (Varian Medical Systems, Palo Alto, CA). Three-dimensional conformal radiotherapy (3D-CRT) or intensity-modulated radiation therapy (IMRT) treatment planning was performed based on conventional 3DCT or the average intensity projections (AIP) for lung SBRT.

### Online Image Acquisition

On the linear accelerator, the patients were aligned according to skin tattoos using an in-room laser system. The CBCT images were acquired with the gantry-mounted onboard imager (Varian Medical Systems, Palo Alto, CA). The first CBCT image was acquired immediately after setup. The scan time was approximately 60 s, and approximately 650 2D kV images were captured during the full 360° rotation. CBCT images were reconstructed using a thickness of 2.5 mm. The CBCT scan was rigidly registered to the planning CT. An automatic registration of the bony anatomy was performed using a user-defined region of interest including the spinal cord. The registration was evaluated by the radiation therapists and manually corrected if necessary. Then, the registered CBCT images were automatically transferred to the Eclipse treatment planning system (Varian Medical Systems).

### Target Volume Contouring

**Figure 1:**
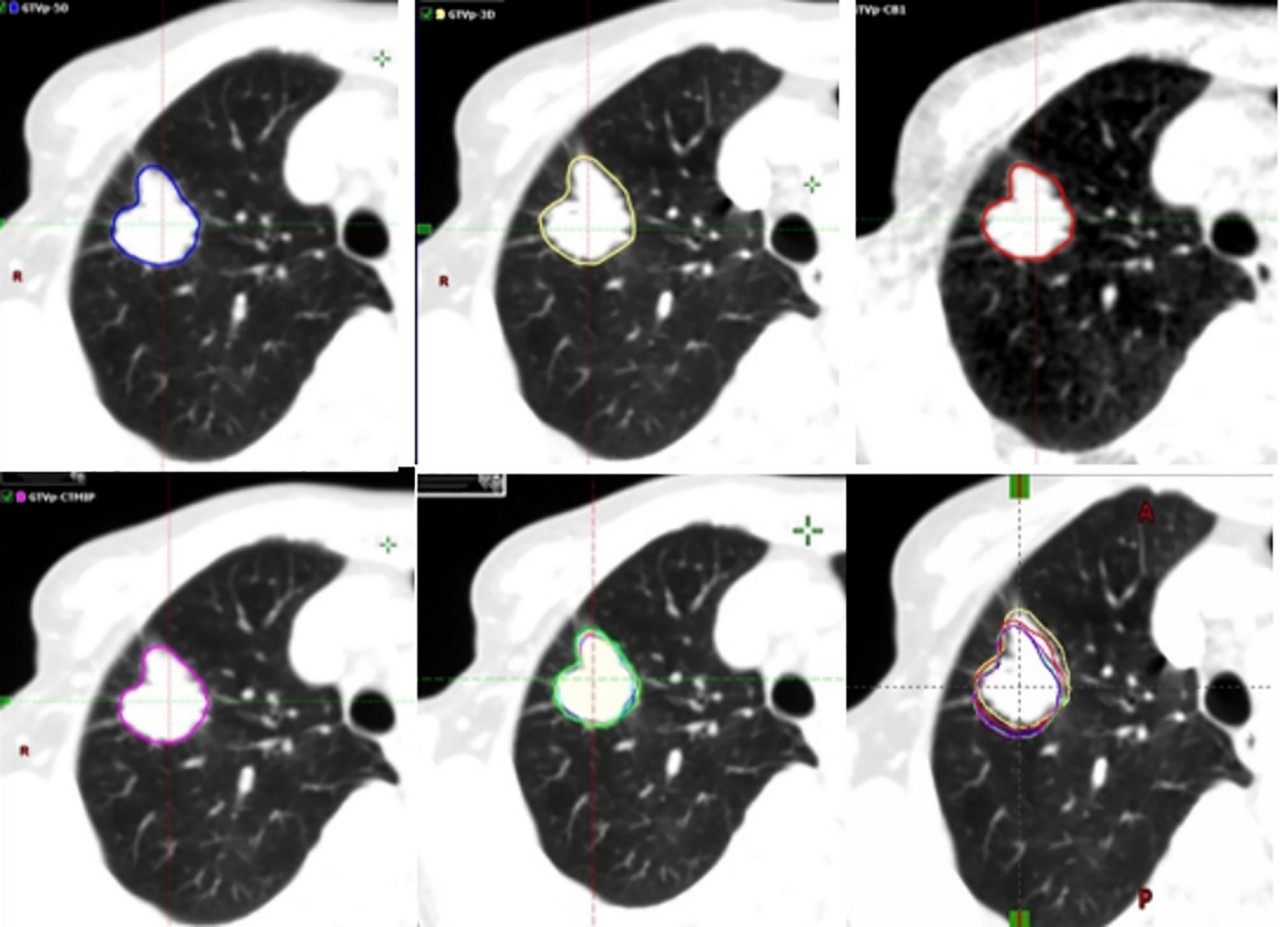
Example (Tumor 132) of the different target volumes. GTV (blue line) contoured based on the end-exhalation of 4DCT, GTV (yellow line) based on 3DCT, GTV (red line) based on CBCT, GTV (magenta line) based on 4DCT MIP, and GTV (green line) derived from ten phases of 4DCT. 3DCT, three-dimensional computed tomography; 4DCT, four-dimensional computed tomography; CBCT, cone-beam computed tomography; GTV, gross tumor volumes; MIP, maximum intensity projection.

GTV-3D images and GTVs were contoured based on 3DCT images and each of the 10 4DCT phases. Internal GTV in the 10 phases (IGTV-10) was generated using the 4D tool. IGTV-MIP and IGTV-CBCT were contoured based on the MIP of 4DCT and CBCT images. All contours were performed by an experienced radiation oncologist using the same contouring protocol, as follows: 1) GTVs were delineated using a standard lung gray-scale window setting in the Aria Eclipse environment (Varian Medical Systems) (25); 2) the use of the standard mediastinum window was allowed for information purposes to avoid the inclusion of adjacent vessels and mediastinal or chest wall structures; and 3) blurring in the periphery of the tumor, representing the “partial volume effect” and “partial projection effect for moving objects”, was included in the GTVs (28). Another radiation oncologist reviewed all contours and rectifed the contour if necessary. The GTVs contoured on the basis of 3DCT, CBCT, end-exhalation, MIP, and the 10 phases of 4DCT of the 132 tumors are shown in **Figure 1**.

### Tumor Motion

The coordinates in the left–right (LR), anterior–posterior (AP), and cranial–caudal (CC) directions of the center of mass (COM) of the GTVs in the 10 phases of 4DCT were measured. The peak-to-peak displacement of the COM in the three directions was calculated based on the coordinates, representing the tumor motion. The 3D motion vector (vector) of the COM was calculated as follows:


$$3\text{D vector}=\sqrt{LR^{2}+AP^{2}+CC^{2}}$$

### Target-Related and Clinical Factors

Target-related factors included the size, location (lobes, abutment relation, and zones), and 3D motion of the target. The size of GTV derived from the end-exhalation of 4DCT was used to represent the size of the target. The abutment relationship referred to solitary pulmonary tumors, and tumors adjacent to the chest wall, the mediastinum, or the diaphragm. Zoning referred to the interior, intermediate, and lateral third zones of the ipsilateral lung. Clinical factors included patient sex, age, body mass index (BMI), body surface area (BSA), KPS, smoking history, pathology, and presence or absence of coexisting pulmonary disease, heart disease, hypertensive disease, or diabetes.

### Dice Similarity Coefficient (DSC)

The DSC of volumes A and B was defined as the ratio of the volume of their intersection to their average volume, with a value of 1 indicating identical volumes and 0 indicating no overlap of the two volumes. It is calculated using the following formula (27, 29):


$$DSC\left(A,B\right)=\frac{2\left(A\cap B\right)}{A+B}$$

The inter-quartile range (IQR) was used to assign the DSCs of IGTV-MIP and IGTV-10 and the DSCs of IGTV-CBCT and IGTV-10 into a qualified group and an unqualified group. The upper quartile was chosen to be the critical value, and the two values were 0.9 and 0.75 for the DSCs of IGTV-MIP and IGTV-10, and the DSCs of IGTV-CBCT and IGTV-10, respectively. A DSC value equal to or more than the critical value was considered as qualified.

### Statistical Analysis

Multiple-logistic regression models were used to explore the risk factors for DSC of IGTV-MIP and IGTV-10 and for DSC of IGTV-CBCT and IGTV-10. Backward stepwise regression based on the Akaike information criterion was used to select important variables. Once the model was established, we used it to predict risk, and the effect of the prediction was presented using a receiver operating characteristic (ROC) curve and the area under the curve (AUC). Individuals’ characteristics were described and grouped by the DSC on IGTV-MIP and IGTV-10 and the DSC on IGTV-CBCT and IGTV-10. Variables were described using means [standard deviations (SD)], medians [IQR], and numbers (%), as appropriate. Differences in these variables were assessed by a two-sample *t* test, Wilcoxon rank-sum test, and X^2^ or Fisher exact test, as appropriate. All analyses were performed using R, version 4.0.4 (R Foundation for Statistical Computing, Vienna, Austria). Hypothesis tests were two-sided, and we considered *p* < 0.05 to be statistically significant.

## Results

The mean tumor motion amplitudes were 1.6 ± 1.2 mm, 2.2 ± 1.5 mm, 5.5 ± 5.2 mm, and 6.5 ± 5 mm in the LR, AP, CC, and 3D directions, respectively. The range of motion amplitude was 0.1-11.4 mm, 0.3-11 mm, 0.1-27.1 mm, and 0.6-27.3 mm in the LR, AP, CC, and 3D directions, respectively.

The mean size of the GTV-3D, GTV-end-expiration (GTV-EE), IGTV-CBCT, IGTV-MIP, and IGTV-10 for all patients was 8.78 ± 12.15 cm^3^, 8.58 ± 11.7 cm^3^, 9.94 ± 13.51 cm^3^, 11.2 ± 15.08 cm^3^, and 13.37 ± 17.11 cm^3^, respectively. The size of the IGTV-10 was greater than the other target volumes (all *p* < 0.001). The size of the IGTV-MIP was larger than that of the IGTV-CBCT (*p* < 0.001). There was no significant difference in volume between the GTV-3D and GTV-EE (*p* = 0.122). The mean size ratios of the GTV-3D, GTV-EE, IGTV-CBCT, and IGTV-MIP compared to the IGTV-10 for all patients were 0.63 ± 0.17, 0.6 ± 0.16, 0.71 ± 0.21, and 0.8 ± 0.14, respectively. The mean size ratio of the IGTV-CBCT to the IGTV-MIP was 0.91 ± 0.26.

**Table 1** presents the baseline characteristics of study variables by the DSC of IGTV-MIP and IGTV-10 and the DSC of IGTV-CBCT and IGTV-10. The qualified group showed a lower 3D motion and larger GTV-EE size in the DSC of IGTV-MIP and IGTV-10, while the unqualified group showed a lower 3D motion, larger GTV-EE, and lower KPS in the DSC of IGTV-CBCT and IGTV-10.

**Table 1:** Baseline Characteristics by DSC (IGTV-MIP, IGTV-10) and DSC (IGTV-CBCT, IGTV-10).

Variables	DSC (IGTV-MIP, IGTV-10)		*p* Value	DSC (IGTV-CBCT, IGTV-10)		*p* Value
	Unqualified Group	Qualified Group		Unqualified Group	Qualified Group	
N	152	58		154	56	
Age, years	63.61 (11.79)	64.19 (11.85)	0.751	62.88 (11.96)	66.23 (11.01)	0.066
Male, n (%)	85 (55.92)	29 (50.00)	0.538	79 (51.30)	35 (62.50)	0.199
Smoker, n (%)	56 (36.84)	21 (36.21)	1.000	52 (33.77)	25 (44.64)	0.199
Cancer pattern, n (%)						
Primary adenocarcinoma	85 (55.92)	31 (53.44)		86 (55.84)	30 (53.57)	
Metastatic tumor	48 (31.58)	16 (27.59)		50 (32.47)	14 (25.00)	
Primary squamous cell carcinomas	19 (12.50)	11 (18.97)	0.474	18 (11.69)	12 (21.43)	0.172
Stage, n (%)						
I	71 (46.71)	22 (37.93)		68 (44.16)	25 (44.64)	
II	3 (1.97)	1 (1.72)		2 (1.30)	2 (3.57)	
III	7 (4.61)	4 (6.90)		8 (5.20)	3 (5.36)	
IV	71 (46.71)	31 (53.45)	0.669	76 (49.34)	26 (46.43)	0.755
Surgery, n (%)	21 (13.82)	11 (18.97)	0.475	23 (14.94)	9 (16.07)	1.000
Abutment, n (%)						
Solitary pulmonary	115 (75.66)	38 (65.51)		110 (71.43)	43 (76.79)	
Adhesion to chest wall	23 (15.12)	12 (20.69)		27 (17.53)	8 (14.29)	
Adhesion to diaphragm	7 (4.61)	4 (6.90)		7 (4.55)	4 (7.14)	
Adhesion to mediastinum	7 (4.61)	4 (6.90)	0.530	10 (6.49)	1 (1.78)	0.442
Position, n (%)						
LUL	42 (27.63)	19 (32.76)		42 (27.27)	19 (33.93)	
LLL	32 (21.05)	11 (18.97)		30 (19.48)	13 (23.21)	
RUL	24 (15.79)	12 (20.69)		24 (15.58)	12 (21.43)	
RML	18 (11.85)	6 (10.34)		21 (13.64)	3 (5.36)	
RLL	36 (23.68)	10 (17.24)	0.748	37 (24.03)	9 (16.07)	0.251
Zoning, n (%)						
Interior	30 (19.74)	13 (22.41)		30 (19.48)	13 (23.21)	
Lateral	73 (48.02)	25 (43.11)		72 (46.75)	26 (46.43)	
Intermediate	49 (32.24)	20 (34.48)	0.807	52 (33.77)	17 (30.36)	0.808
3D motion, mm	5.02 [3.27, 9.81]	3.98 [2.14, 6.76]	0.025	4.96 [3.43, 9.91]	3.80 [1.95, 6.37]	0.005
GTV-EE size, cm^3^	3.60 [1.60, 7.30]	6.55 [3.00, 22.93]	<0.001	3.25 [1.63, 6.83]	10.15 [3.75, 25.41]	<0.001
KPS, scores	85.92 (5.80)	84.10 (6.74)	0.052	85.96 (5.77)	83.93 (6.79)	0.031
BMI, kg/m^2^	24.50 (3.03)	24.07 (3.23)	0.364	24.40 (2.94)	24.33 (3.49)	0.877
BSA, cm^2^	1.76 (0.18)	1.77 (0.14)	0.839	1.76 (0.16)	1.794 (0.19)	0.148
Diabetes, n (%)	14 (9.21)	4 (6.90)	0.795	15 (9.74)	3 (5.36)	0.469
Hypertension, n (%)	37 (24.34)	12 (20.69)	0.706	38 (24.68)	11 (19.64)	0.563
Cardiopathy, n (%)	37 (24.34)	12 (20.69)	0.706	41 (26.62)	8 (14.29)	0.092
Pulmonary disease, n (%)	66 (43.42)	24 (41.38)	0.911	68 (44.16)	22 (39.29)	0.636

DSC (IGTV-MIP, IGTV-10) is considered as qualified when it is equal or more than 0.9. DSC (IGTV-CBCT, IGTV-10) is considered as qualified when it is equal to or more than 0.75. Continuous variables are presented as means (SD) or medians [IQR]. Categorical variables are presented as n (%). BMI, body mass index; BSA, body surface area; DSC, Dice’s similarity coefficient; IGTV-10, internal gross target volume from 10 phases of four-dimensional computed tomography; IGTV-CBCT, internal gross target volume-cone beam computed tomography; IQR, interquartile range; LUL, left upper lobe; LLL, left lower lobe; RUL, right upper lobe; RML, right middle lobe; RLL, right lower lobe; SD, standard deviation.

**Figure 2:**
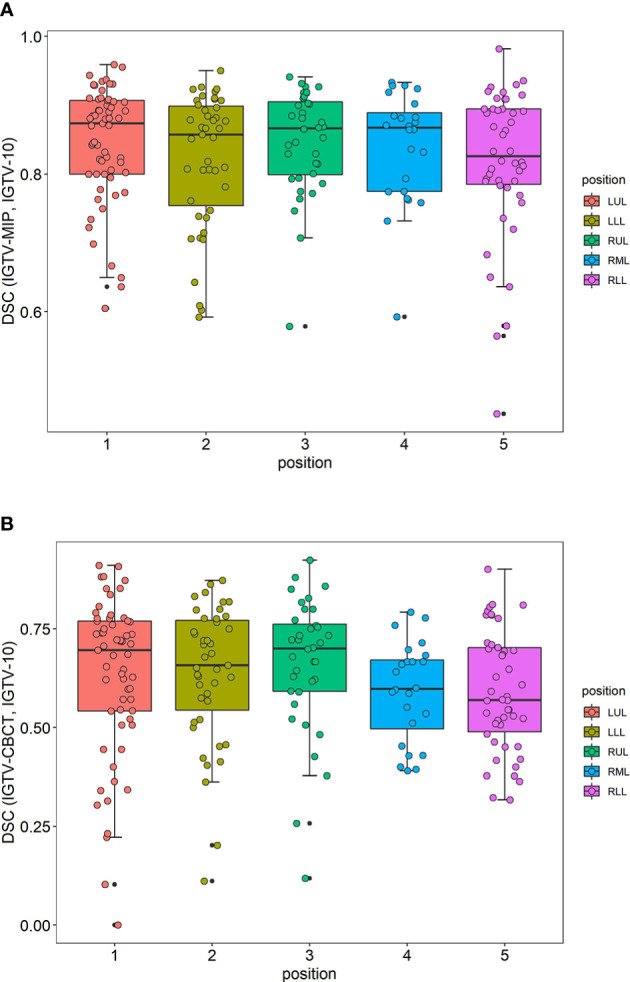
Distributions of DSC. **(A)** The distribution of DSC (IGTV-MIP, IGTV-10) grouped by position of cancer. **(B)** The distribution of DSC (IGTV-CBCT, IGTV-10) grouped by position of cancer. DSC, Dice’s similarity coefficient; IGTV-10, internal gross target volume from 10 phases of four-dimensional computed tomography; IGTV-CBCT, internal gross target volume-cone beam computed tomography; LUL, left upper lobe; LLL, left lower lobe; RUL, right upper lobe; RML, right middle lobe; RLL, right lower lobe.

The distribution of the DSC of IGTV-MIP and IGTV-10 grouped by position of the cancer is included in **Figure 2A**. The distribution of the DSC of IGTV-MIP and IGTV-10 was a skewed distribution, and tumors in the right lower lobe had a worse DSC. The distribution of the DSC of IGTV-CBCT and IGTV-10 grouped by the position of the cancer is included in **Figure 2B**. A skewed distribution was also found, and worse DSCs were observed in the right middle lobe and right lower lobe.

**Table 2** presents the standardized odds ratios (ORs) and 95% confidence intervals (95% CIs) between study variables and the DSC of IGTV-MIP and IGTV-10. The results show that female sex, greater BSA, and larger GTV-EE were protective factors with ORs of 3.89 (1.45, 11.17), 1.97 (1.10, 3.65), and 2.20 (1.57, 3.20), respectively. KPS, BMI, and 3D motion were considered risk factors with ORs of 0.70 (0.50, 0.98), 0.58 (0.35, 0.95), and 0.65 (0.42, 0.95), respectively.

**Table 2:** Odds ratios and 95% Confidence intervals of DSC (IGTV-MIP, IGTV-10).

Variables	Standard OR (95% CI)	Estimate	Std. Estimate	*p* Value
Female	3.89 (1.45, 11.17)	1.358	1.358	0.009
BSA	1.97 (1.10, 3.65)	3.995	0.677	0.026
KPS	0.70 (0.50, 0.98)	-0.057	-0.350	0.038
BMI	0.58 (0.35, 0.95)	-0.175	-0.539	0.034
3D motion	0.65 (0.42, 0.95)	-0.086	-0.435	0.033
GTV-EE size	2.20 (1.57, 3.20)	0.065	0.788	<0.001

3D, three-dimensional; BMI, body mass index; BSA, body surface area; CI, confidence interval; DSC, Dice’s similarity coefficient; GTV-EE, gross target volume end of expiration; IGTV-10, internal gross target volume from 10 phases of four-dimensional computed tomography; IGTV-MIP, internal gross target volume maximum intensity projection; KPS, Karnofsky Performance Status; OR, odds ratio; SD, standard deviation.

**Table 3** shows standardized ORs and 95% CIs between selected variables and the DSC of IGTV-CBCT and IGTV-10. The results show that older age and larger GTV-EE were protective factors with ORs of 1.66 (1.09, 2.65) and 3.89 (2.41, 6.83), respectively. Adhesion to the heart, 3D motion, and cardiopathy were considered risk factors with ORs of 0.06 (0.00, 0.57), 0.45 (0.26, 0.75), and 0.32 (0.11, 0.83), respectively. Adhesion to the parietal pleura, BSA, and pulmonary disease showed marginal significance; however, if there was a larger sample size, these variables may have shown a statistically significant difference.

**Table 3:** Odds ratios and 95% confidence intervals of DSC (IGTV-CBCT, IGTV-10).

Variables	Standard OR (95% CI)	Estimate	Std. Estimate	*p* Value
Age	1.660 (1.086, 2.652)	0.043	0.509	0.025
**Abutment**				
Solitary pulmonary	reference	–	reference	–
Adhesion to chest wall	0.385 (0.116, 1.108)	-0.954	-0.954	0.095
Adhesion to diaphragm	0.430 (0.033, 4.713)	-0.843	-0.843	0.507
Adhesion to mediastinum	0.057 (0.002, 0.567)	-2.865	-2.865	0.037
BSA	1.409 (0.960, 2.098)	2.023	0.343	0.084
3D motion	0.454 (0.257, 0.749)	-0.157	-0.790	0.004
GTV-EE size	3.891 (2.412, 6.834)	0.113	1.356	<0.001
Pulmonary disease	0.468 (0.204, 1.030)	-0.760	-0.760	0.065
Cardiopathy	0.316 (0.106, 0.827)	-1.153	-1.153	0.026

3D, three-dimensional; BSA, body surface area; CI, confidence interval; DSC, Dice’s similarity coefficient; GTV-EE, gross target volume end of expiration; IGTV-10, internal gross target volume from 10 phases of four-dimensional computed tomography; IGTV-CBCT, internal gross target volume-cone beam computed tomography; OR, odds ratio.

**Figure 3:**
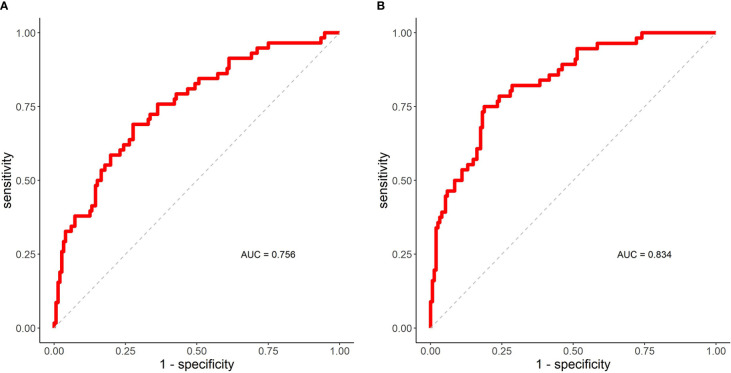
ROC curves and AUC. **(A)** The ROC curve and AUC of DSC (IGTV-MIP, IGTV-10) prediction model. **(B)** The ROC curve and AUC of DSC (IGTV-CBCT, IGTV-10) prediction model. AUC, area under the curve; DSC, Dice’s similarity coefficient; IGTV-10 indicates internal gross target volume from 10 phases of four-dimensional computed tomography; IGTV-CBCT, internal gross target volume-cone beam computed tomography; IGTV-MIP, internal gross target volume maximum intensity projection; ROC, receiver operating curve.

The ROC curve and AUC of the DSC of the IGTV-MIP and IGTV-10 prediction models are shown in **Figure 3A**. It shows that AUC was equal to 0.756, which means the prediction effect was good. The ROC curve and AUC of the DSC of the IGTV-CBCT and IGTV-10 prediction models are shown in **Figure 3B**. The AUC was equal to 0.834, representing a good prediction effect.

## Discussion

4DCT and 3DCT images acquired during simulation are usually used to generate treatment target volumes, while the CBCT image acquired before treatment is used to verify the volumes. A thorough understanding of the potential relationship between the volumes derived from 4DCT, 3DCT, and CBCT images may contribute to increased accuracy of SBRT. Previous reports have merely clarified the impact of target characteristics on the volumes on 4DCT, 3DCT, and CBCT images in phantom studies and small patient collectives. We systematically investigated the influence of target-related and clinical characteristics on the volumes in a large study population.

When comparing the difference in size between IGTV-MIP and IGTV-10, we found that IGTV-MIP size was, on average, 20% smaller than IGTV-10 size. This finding was consistent with that of previous studies (10, 14, 15). Borm et al. (15) showed that 4DCT MIP-based ITVs were 20.2% smaller on average than 10-phase 4DCT ITVs. Muirhead et al. (10) showed a mean ITV reduction of 19% in ITV-10-phase volumes compared to ITV-MIP volumes. However, some reports are not consistent with our results (7, 11). Ge et al. (7) reported that the ITV derived from MIP showed an underestimation of approximately 10% compared with that of 10-phase 4DCT. They found a mean volumetric difference between PTV-MIP and 4D PTV-10 of 7% ± 5%. These inconsistencies suggest that some potential influencing factors might have led to the differences in size between IGTV-MIP and IGTV-10. Previous studies have reported that the tumor size, motion amplitude, and abutment relationship might have an influence on this difference (7, 10, 14, 15). Our study showed the tumor size had a positive correlation with the size ratio of IGTV-MIP to IGTV-10 (*r* = 0.327, *p* < 0.001), while tumor motion had a negative correlation to the ratio (*r* = -0.207, *p* = 0.003). Additionally, we found that tumor location and smoking history had an influence on this difference (*p* = 0.013 and 0.043). Other characteristics (for example, abutment relationship, pulmonary surgery, cardiopulmonary disease, primary or metastatic carcinoma, and so on) had no significant influence on the difference in size.

Further analysis found a mean DSC of IGTV-MIP and IGTV-10 of 0.84 ± 0.09. We set the cutoff value as the third quartile of the DSC (0.9) to evaluate the pros and cons of the similarity between IGTV-MIP and IGTV-10 and analyzed the impact of target-related and clinical factors on the threshold. Our finding that larger targets with small tumor motion in the 3D direction had a better DSC than small targets or those with larger tumor motion supports previously published studies (8, 15). However, the abutment relationship was not significantly correlated with the DSC in our study, even though the DSC for tumors adjoined to the diaphragm tended to have a poor DSC.

Additionally, female sex, BSA, BMI, and KPS were significantly associated with the DSC of IGTV-MIP and IGTV-10. Sex was an important influencing factor (std. estimate = 1.358), and female patients had a better DSC than male patients. We believe that this is because female patients tended to have a smaller tumor motion and BMI than male patients. It is possible that a larger BMI (std. estimate = -0.539) reduced the DSC of IGTV-MIP and IGTV-10 because BMI had a negative impact on the sharpness of MIP images. The ROC curve and AUC of the DSC of the IGTV-MIP and IGTV-10 prediction models showed that the AUC was equal to 0.756, indicating a good prediction effect. The significance of clinical characteristics should be highlighted when using IGTV-MIP in SBRT.

In this study, on average, IGTV-CBCT size was 29% smaller than IGTV-10 size and 9% smaller than IGTV-MIP size, which was in accordance with results reported by other authors. Vergalasova et al. (30) reported that the IGTV derived from free-breathing CBCT showed a volume underestimation of 40.1% for smaller tumors and 24.2% for larger tumors compared to the 4DCBCT-based IGTV. Liu et al. (25) found the medium IGTV-CBCT was, on average, approximately 11.8% smaller than the IGTV based on end-inhalation and end-exhalation phases. Wang et al. observed that the IGTV from CBCT was 3.1-9.3% smaller than that derived from 4DCT MIP. However, Wang et al. (19) reported that the difference in size between IGTVs derived from CBCT and 4DCT 10-phases was within 8%, which was a far smaller difference than that noted in our result (29%). Some studies (30, 31) have shown that irregular breathing patterns might lead to this misinterpretation.

Additionally, Wang et al. (19) hypothesized that the characteristics of the target and the patient might have an impact on the CBCT target volume. They concluded that the location of the tumor was a major source of discrepancy between ITV-CBCT and ITV-10. We believe that the relatively small number of patients (n=71) included in their study might have impacted their results. For this reason we included a larger number of tumors (n=210) and we investigated the clinical features to more accurately assess the influence of the target and patient characteristics on the DSC of IGTV-CBCT and IGTV-10.

The mean DSC of IGTV-MIP and IGTV-10 was 0.64 ± 0.17. The cut-off value was still defined as the third quartile of the DSC of IGTV-CBCT and IGTV-10 (0.75). Multivariate analysis showed that the tumor abutment relationship was an important factor impacting the DSC, particularly for tumors adjoined to the mediastinum (heart) where the DSC was worse. Additionally, we found that combining cardiopulmonary disease and larger tumor motion might reduce the DSC of the IGTV-CBCT and IGTV-10, while larger tumor size, age, and BSA might increase the DSC. The AUC of the DSC of the IGTV-MIP and IGTV-10 prediction models was 0.834 and represents a good prediction effect. Although a PTV margin is used in clinical practice, this finding indicated that an extra margin might be required to account for the discrepancy between IGTV-CBCT and IGTV-10 derived from the target-related and patient characteristics.

We also evaluated the difference in size between GTV-3D and IGTV-10 and between GTV-EE and IGTV-10 among a greater number of patients because previous studies were usually based only on a few cases. The size of the GTV-3D was 47% smaller than the IGTV-10 size, and the GTV-EE size was 50% smaller. These results were consistent with those of previous studies (26, 32). Some tumor-related and patient features may have an impact on these differences.

It should be noted that all the contouring was performed by one oncologist to avoid interobserver variability. Although a systematic intra-observer variability may be inevitable, using an oncologist who was experienced in contouring and strict contouring criteria contributed to reduced variability. Additionally, we adopted the first CBCT image to remove the impact of the tumor reduction. But, the first CBCT may not represent the interfraction variability. The amplitude and baseline of respiration motion might change throughout the treatment (21). There would be an inherent variation between IGTV-10 derived from 4D CT and IGTV-CBCT derived from treatment CBCT, which may deduce incorrection conclusions.

## Conclusion

In a large sample, we identified the discrepancy between IGTV-MIP and IGTV-10, and between IGTV-CBCT and IGTV-10. The target-related factors (such as tumor motion and size) showed significant influences on the discrepancy. Moreover, several clinical factors could significantly influence the discrepancy between IGTVs derived from 4DCT, 4DCT MIP, and CBCT. The prediction models of the DSC of IGTVs derived from 4DCT and CBCT showed good predictive value. The clinical factors should be considered when using MIP images for defining the ITV and when using CBCT images for verifying the treatment targets.

## Data Availability Statement

The original contributions presented in the study are included in the article/supplementary material. Further inquiries can be directed to the corresponding author.

## Ethics Statement

The studies involving human participants were reviewed and approved by Shandong Cancer Hospital and Institute ethics board. Written informed consent for participation was not required for this study in accordance with the national legislation and the institutional requirements. Written informed consent was obtained from the individual(s) for the publication of any potentially identifiable images or data included in this article.

## Author Contributions

FL and JL contributed to the study design, the delineation and writing the manuscript, the patient enrollment. TTZ and YQ participated the data statistics and analysis and writing the manuscript. XS and ZC contributed to the patient enrollment. TZ participated in the study design and data statistics and analysis. All authors read and approved the final manuscript.

## Funding

National Natural Science Foundation of China (817732870); Beijing Medical Award Foundation (YXJL-2020-0785-0616); Taishan Scholars Program of Shandong Province (NO.ts 20190982); Wu Jieping Medical Foundation (320.6750.2021-02-79).

## Conflict of Interest

The authors declare that the research was conducted in the absence of any commercial or financial relationships that could be construed as a potential conflict of interest.

## Publisher’s Note

All claims expressed in this article are solely those of the authors and do not necessarily represent those of their affiliated organizations, or those of the publisher, the editors and the reviewers. Any product that may be evaluated in this article, or claim that may be made by its manufacturer, is not guaranteed or endorsed by the publisher.
